# NF-kappaB Signaling in Chronic Inflammatory Airway Disease

**DOI:** 10.3390/biom5031266

**Published:** 2015-06-26

**Authors:** Michael Schuliga

**Affiliations:** Lung Health Research Centre (LHRC), Department Pharmacology and Therapeutics, University of Melbourne, Grattan St., Parkville 3010, Victoria, Australia; E-Mail: schuliga@unimelb.edu.au; Tel.: +61-8344-8508; Fax: +61-8344-0241

**Keywords:** airway smooth muscle, allergic asthma, alveolar macrophages, cigarette smoke, eosinophils, epithelium, emphysema, histone deacetylase, phosphoinositide 3 kinase-delta (PI3K-δ), sirtuins

## Abstract

Asthma and chronic obstructive pulmonary disease (COPD) are obstructive airway disorders which differ in their underlying causes and phenotypes but overlap in patterns of pharmacological treatments. In both asthma and COPD, oxidative stress contributes to airway inflammation by inducing inflammatory gene expression. The redox-sensitive transcription factor, nuclear factor (NF)-kappaB (NF-κB), is an important participant in a broad spectrum of inflammatory networks that regulate cytokine activity in airway pathology. The anti-inflammatory actions of glucocorticoids (GCs), a mainstay treatment for asthma, involve inhibition of NF-κB induced gene transcription. Ligand bound GC receptors (GRs) bind NF-κB to suppress the transcription of NF-κB responsive genes (*i.e.*, transrepression). However, in severe asthma and COPD, the transrepression of NF-κB by GCs is negated as a consequence of post-translational changes to GR and histones involved in chromatin remodeling. Therapeutics which target NF-κB activation, including inhibitors of IκB kinases (IKKs) are potential treatments for asthma and COPD. Furthermore, reversing GR/histone acetylation shows promise as a strategy to treat steroid refractory airway disease by augmenting NF-κB transrepression. This review examines NF-κB signaling in airway inflammation and its potential as target for treatment of asthma and COPD.

## 1. Introduction

Asthma and chronic obstructive pulmonary disease (COPD) are obstructive lung diseases characterized by a limitation of airflow caused by the narrowing of bronchioles. COPD also comprises irreversible breakdown of lung tissue (emphysema). Asthma and COPD contribute to ~0.25 and 3 million deaths per year worldwide, respectively [1,2]. Whilst differing in etiology, asthma and COPD share clinical symptoms including shortness of breath, wheezing, coughing and sputum production [3]. Inflammation is unequivocally an important component of these diseases, with lung inflammatory cell profiles varying considerably depending on disease phenotype and which change upon disease exacerbation [4,5,6]. Anti-inflammatory glucocorticoids are a mainstay therapy for asthma, but are ineffective as a treatment for those with severe asthma (5%–10% of asthmatics) or COPD [7]. For these patients, there remains an unmet need for alternative therapies which target airway inflammation, its underlying causes, or reverse glucocorticoid (GC) insensitivity.

The therapeutic efficacy of GCs is largely contributed by GC inhibition of the transcription factor, nuclear factor (NF)-kappaB (NF-κB) [8]. The latter is comprised of homo- or heterodimers of NF-κB1/p50, NF-κB2/p52, RelA/p65, Rel/c-Rel, and RelB [9]. The activation of NF-κB occurs when it dissociates from IκBα, the negative regulator of NF-κB which is degraded in the process. NF-κB activation involves IκBα phosphorylation catalyzed by IκB kinase (IKK) [10]. Liberation from IκB allows NF-κB to translocate into the nucleus where it induces gene transcription by binding to the promoter of NF-κB responsive genes. NF-κB has an important role in airway pathology by regulating the expression of cytokines, chemokines and cell adhesion molecules (CAMs) [11,12,13,14]. These inflammatory mediators influence the type and quantity of inflammatory cells that infiltrate airway tissue in chronic airway obstructive diseases. NF-κB activation in asthma and COPD occurs largely in response to inflammatory mediators such as interleukin (IL)-1β and tumour necrosis factor (TNF)-α or elicited by the activation of toll like receptors (TLRs) during bacterial or viral-exacerbation [15]. NF-κB signaling is redox regulated, being influenced by an oxidant/antioxidant imbalance that occurs in the airways in disease [16]. The anti-inflammatory actions of GCs involve the binding of ligand bound GC receptor (GR) to NF-κB in a process called “transrepression” [17,18]. This GR binding in effect inhibits NF-κB-augmented transcriptional activation.

As multiple signal transduction pathways in inflammation converge on the NF-κB/IκBα complex, various strategies that target NF-κB signaling have been considered for asthma and COPD treatment. Therapeutics that target NF-κB/IκBα complex dissociation or NF-κB/DNA binding may potentially treat GC-insensitive airway disease [15]. However as the NF-κB/IκBα complex is ubiquitously expressed, there are issues with selectivity and potential side effects. Another approach in the treatment of steroid refractory airway disease is to reverse GC-insensitivity. GC resistance in severe asthma and COPD involves prolonged acetylation of the GR and histones, which prevent binding of GR to NF-κB [7]. Theophylline-like drugs, phosphoinositide 3 kinase-delta (PI3K-δ) inhibitors and non-antibiotic macrolides which reduce GR/histone acetylation in the disease state are promising treatments for steroid refractory asthma and COPD [19].

This paper will give an overview of NF-κB signaling in chronic airway disease, providing an update of research in an area last thoroughly reviewed in 2009 [15]. Progress and areas of active research since include: (i) a greater understanding of NF-κB signaling in both cellular and animal models of asthma and COPD, particularly in airway structural cells; (ii) greater characterization of the effects of NF-κB inhibitors in experimental models of airway disease; (iii) new insights into the protective effects of NF-κB signaling in cigarette smoke-evoked pulmonary inflammation, which cautions against the use of NF-κB inhibitors as a COPD treatment; and (iv) the pre-clinical investigations into alternative treatments which target HDAC activity to modulate NF-κB transcriptional activation/GC-sensitivity.

## 2. Asthma and COPD

### 2.1. Overview of Asthma and COPD

Asthma and COPD are a set of heterogeneous airway obstructive diseases characterized by varying patterns of airway inflammation and airway wall remodeling (AWR). The prevalence of asthma is between 5% and 10% globally affecting most age groups [20]. The prevalence of COPD (stage II or higher) is ~10%, being much higher in older people with a history of smoking [21]. Asthma is more likely to be caused by an allergic response whereas exposure to cigarette smoke is the major etiologic factor that contributes to the pathogenesis of COPD. Patients with severe asthma (5%–10% of asthmatics) or COPD remain symptomatic despite glucocorticoid treatment [3]. T-helper(Th)2 lymphocyte-driven allergic asthma is the most common asthma phenotype, albeit other phenotypes exist [22]. Airway inflammation in asthma varies with phenotype, comprising increased numbers of Th2 lymphocytes, mast cells and eosinophils to an inflammation more neutrophilic with greater Th1 lymphocyte involvement [5]. COPD is characterized by neutrophilic inflammation of the small airways [5], ranging from airway to emphysema predominant phenotypes [22]. AWR in asthma and COPD comprise structural changes to the tissue of airway wall including increased mucous production, collagen deposition and airway smooth muscle (ASM) hyperplasia/hypertrophy. AWR contributes to airway reactivity and is considered to occur through injury and a dys-regulated repair processes linked to inflammation.

### 2.2. Evidence of NF-κB in Asthma

NF-κB signaling in asthma is evidenced by increased NF-κB nuclear localization or DNA binding, IκB phosphorylation or degradation, and IKK-β expression in the airway tissue of asthmatics [23,24]. Increased NF-κB nuclear binding or staining is also detected in the inflammatory cells of the induced sputum of asthmatics [23]. Additional support for a role of NF-κB in asthma comes from a range of *in vivo* studies showing increased NF-κB activation in airway tissue and inflammatory cells following intranasal challenge with allergen (e.g., ovalbumin or house dust mite (HDM) extract) [25,26,27], endotoxin [28], or microbial infection [29]. In recombinant mice deficient in intermediates of NF-κB signaling, including NF-κB [30] and IκBα [25], allergen-induced airway inflammation is attenuated. Furthermore, NF-κB activation in the airways of allergen-challenged mice is attenuated by TLR2 or TLR4 gene deletion, suggesting that the innate immune system contributes to NF-κB signaling in asthma [31,32]. The clinical evidence for an effect of GCs on NF-κB gene expression and signaling in asthma is rather more mixed. Inhaled budesonide has been observed to decrease the levels of activated NF-κB, along with cytokines and CAMs, in human airway wall tissue [33,34]. However, inhaled fluticasone, another GC used in asthma therapy, was reported to have little effect on NF-κB activation in the tissue of the human airway wall despite its use improving lung function and reducing eosinophilic infiltration [35].

### 2.3. Evidence of NF-κB in COPD

As in asthma, higher levels of activated NF-κB are observed in the bronchial biopsies and inflammatory cells of COPD individuals [36,37]. IκBα levels in lung tissue of smokers and COPD patients are also less than non-smoking healthy individuals [38]. IKK activity in peripheral blood mononuclear cells (PBMCs) obtained from donors with COPD or smokers is also higher than controls [39]. Furthermore, the inhibitory effects of GCs on TNFα-stimulated IL-8 release from PBMCs of COPD and smokers is less than asthmatics and controls [39]. Additionally, the neutrophils in the sputum of COPD donors show increased NF-κB signaling following exposure to cigarette smoke (CS) extract [40]. In an *in vivo* COPD model, CS-extract exposure increases the levels of NF-κB and its recruitment to the promoters of inflammatory genes in the mouse lung [41]. *In vivo*, the pharmacological or genetic targeting of NF-κB intermediates including IKK has been observed to attenuate airway inflammation in response to CS extract or lipopolysaccharide (LPS), a component of CS [42,43,44]. However, a detailed *in vivo* study has also reported that IKK inhibition or gene deletion has little effect on CS-induced airway inflammation [44]. A possible explanation for this deviation may be due to differences in the models (*i.e.*, rats *versus* mice). Furthermore, certain strains of mice appear to be resistant to the effects of CS on airway inflammation [45].

## 3. Role of NF-κB in Airway Cells in Disease

In asthma and COPD, infiltrating inflammatory cells produce an array of inflammatory mediators, including cytokines, chemokines and cell adhesion molecules (CAMs), which act by autocrine and paracrine mechanisms to not only regulate the function of inflammatory cells, but also epithelial, smooth muscle, fibroblast and endothelial cells. The latter structural cells are also potent producers of inflammatory mediators [11,12,13,14]. NF-κB has a central role in regulating the expression of inflammatory genes in airway cells (summarized in Table 1), including those discussed below.

**Table 1:** Inflammatory genes regulated by NF-κB in airway cells types.

Cell Type	Genes
Lymphocytes (Th1/Th2)	Eotaxin-1, regulated and activation normal T cell expressed and secreted (RANTES), Th1 [interferon (IFN)-gamma and interleukin (IL)-2], Th2 [IL-4, IL-5 and IL-13] [46]
Eosinophils	TNF-α, IL-8, intercellular adhesion molecule (ICAM)-1 and leukocyte function-associated antigen-1 (LFA-1) [47,48]
Neutrophils	IL-8, granulocyte macrophage-colony-stimulating factor (GM-CSF), IL-1Ra [49]
Macrophages	Monocyte chemotactic protein-1 (MCP-1), IL-8 and growth-regulated oncogene-α (GROα) [50,51]
Epithelial cells	Thymic stromal lymphopoietin (TSLP), ICAM-1, vascular adhesion molecule (VCAM)-1, IL-8, IL-6, GM-CSF, chemokine (C-X-C motif) ligand (CXCL)1, RANTES, GROα, MCP-1, eotaxin-1 and MUC5AC [52,53,54,55]
Smooth muscle	TSLP, CD38, VCAM-1, ICAM-1, cyclooxygenase-2, IL-6, IL-8, CXCL10 (a chemoattractant for mast cells), GM-CSF, RANTES, MCP-1, GROα, neutrophil-activating protein-2 (NAP-2) and epithelial neutrophil activating peptide 78 (ENA-78) [56,57,58,59,60,61,62,63]

### 3.1. Lymphocytes

Lymphocytes have an important immunoregulatory role in asthma. In eosinophilic allergic asthma, naïve T cells maturate into Th2 cells that produce IL-4, IL-5 and IL-13 (in a NF-κB-dependent manner), which stimulate B lymphocyte immunoglobulin (Ig)E production [64]. These processes drive eosinophilic inflammation, which also involves mast cell degranulation. Nonallergic eosinophilic asthma is coordinated by innate lymphocytes including innate lymphoid cells type 2 (ILC2) and natural killer T cells (NKT cells) [65]. Neutrophilic asthma involves Th1 and Th17 lymphocytes [64]. IL-17A, IL-17F and IL-22 are produced mainly by Th17 cells, and increased in the airways in steroid refractory severe asthma [66]. Th17 cytokines have an important role in neutrophil recruitment, and contribute to structural changes in bronchial epithelial and airway smooth muscle (ASM) cells [67]. The actions of IL-17 and/or IL-22 in epithelial mucin production and ASM cell proliferation involve NF-κB signaling [68,69,70].

### 3.2. Eosinophils

Airway inflammation in allergic asthma is characterized by eosinophil infiltration and activation. Eosinophil contact with airway epithelial cells stimulates NF-κB-dependent production of CAMs [48]. In peripheral blood eosinophils from atopic donors, allergen activates NF-κB and subsequent production of cytokines (e.g., TNF-α and IL-8) [47]. Additionally, NF-κB signaling is important in eosinophil survival, mediating the anti-apoptotic effect of autocrine TNF-α [71].

### 3.3. Neutrophils

Neutrophil infiltration in the airway submucosa is a major contribution to airway inflammation in neutrophilic asthma and COPD. IL-8 produced by neutrophils is a NF-κB regulated gene which is an important mediator of neutrophilia [72]. In neutrophilic asthma and COPD, Th17 cells and macrophages respectively coordinate the neutrophilic response [73,74]. Neutrophilic inflammation is associated with steroid resistance [75].

### 3.4. Macrophages

Alveolar macrophages play a key role in orchestrating the inflammatory events which occur in the airways in COPD. Exposure to CS extract stimulates a marked increase in secretion of numerous cytokines by alveolar macrophages. Many of these inflammatory mediators are chemoattractants for neutrophils, increasing their infiltration in the lung/airways, a key event in the pathogenesis of COPD. The molecular basis for CS evoked amplified inflammatory responses within the macrophage involves oxidative stress and subsequent NF-κB activation and signaling [50,51].

### 3.5. Epithelial Cells

Epithelial cells have a pivotal role in airway pathology, being an important interface with the environment [62]. The epithelium orchestrates coordinated responses to various invasive insults by producing a myriad of immune/inflammatory mediators regulated by NF-κB (Table 1). Many of these mediators are chemoattractants, which facilitate the recruitment of inflammatory cells that in turn influence epithelial function. Production of TSLP by bronchial epithelial cells in particular is important for the initiation of allergic airway inflammation through a dendritic cell-mediated T helper 2 response [52]. Co-culture with mast cells or eosinophils stimulates NF-κB regulated cytokine and CAM production in airway epithelial cells [48,76]. The airway epithelium is also a source of mucin, which is overproduced in asthma and COPD. The neutrophil-derived protein factors S100A8 and S100A9 stimulate increased production of mucin in airway epithelial cells via TLR4 activation and subsequent downstream NF-κB signaling [77].

### 3.6. Smooth Muscle Cell

The mechano-contractile properties of the airway smooth muscle cell (ASM) make it the primary effecter of bronchospasm, an acute contraction of the airways in asthma. ASM cells are also involved in persistent tissue structural changes of the airway wall that contribute to airway obstruction in asthma and COPD [78,79,80]. ASM cells also have an important inflammatory role in airway obstructive diseases, being potent producers of growth factors, cytokines and other inflammatory mediators [11]. Their expression in ASM cells is stimulated by cytokines and growth factors produced by inflammatory cells and the epithelium [81]. Thrombin and interleukin-1α (IL-1α) stimulate NF-κB activity in ASM cells in a GC-sensitive manner [82]. In ASM cells, NF-κB regulates numerous genes important in asthma and/or COPD pathology (Table 1). IL-8 hypersecretion by ASM cells of asthmatic donors involves increased NF-κB binding to the IL-8 promoter [60].

## 4. GC-Insensitivity in Severe Asthma and COPD

There has been considerable effort to understand the molecular basis of GC resistance in severe asthma and COPD so that strategies to overcome diminished GC sensitivity can be developed [83,84,85,86]. Part of this phenomenon is contributed by impaired transrepression of NF-κB by GCs [19]. Transrepression is a consequence of reduced histone deacetylase (HDAC) expression and activity. Increased oxidative and nitrative stress triggered by insults such as cigarette smoke, severe inflammation, bacterial colonization and/or viral infection are implicated in HDAC abnormalities [19,87]. Defective HDAC leads to histone hyperacetylation associated with a loosening of chromatin structure, which allows the transcription of otherwise silent inflammatory genes [88,89]. Another consequence of reduced HDAC expression and activity is that ligand-bound GR remains acetylated for longer, preventing its association with NF-κB in the nucleus. Increased GR acetylation in alveolar macrophages of COPD donors impedes binding between NF-κB and GR, contributing to GC-insensitive cytokine production [90]. Post-translational modifications to NF-κB and IκBα by other acetyl transferases and deacetylases may be involved in GC resistance in severe asthma and COPD. The sirtuins, proteins with histone and non-histone protein deacetylase activity, are down-regulated in tissue and inflammatory cells of COPD individuals or CS extract-exposed alveolar macrophages [91]. Decreased sirtuin 1 (Sirt1) activity is implicated in the increased acetylation of NF-κB and subsequent IL-8 release in alveolar macrophages of smokers and COPD individuals [91]. Recently, sirtuin 4 (Sirt4) was shown to protect against CS extract-induced cytokine and CAM expression in pulmonary endothelial cells in a manner that prevents IκBα degradation [92].

## 5. The Effect of GC-Transactivation on NF-κB Signaling

GCs not only regulate NF-κB signaling via transrepression, but also by transactivation mechanisms involving direct DNA binding by ligand-occupied GR dimers. The stimulatory effect of GCs on IκBα gene expression is well established, occurring also in structural airway cells [83,84,93]. A number of other proteins whose gene expression is stimulated by GCs via transactivation also regulate NF-κB signaling. The expression of glucocorticoid induced leucine zipper (GILZ) is up-regulated in airway epithelial cells in response to GCs [83,84,93,94]. GILZ inhibits NF-κB signaling via directly binding to NF-κB subunits [95]. Another gene up-regulated by GCs in airway cells is MKP-1, a critical negative feedback controller, limiting the extent and duration of pro-inflammatory MAPK-driven cellular signalling pathways in airway cells [96,97]. MKP-1, via a mechanism not yet established also inhibits NF-κB signaling in airway epithelial cells [98]. As many of the negative side-effects of long term GC treatment are thought to involve transactivation, there has been considerable interest in the development and therapeutic use of transrepression-selective GCs. However, the inhibitory effects of GC-upregulated IκBα, GILZ and MKP-1 on NF-κB signaling suggest that some of the more potent anti-inflammatory effects of GCs also involve transactivation.

## 6. Targeting NF-κB Signaling in Airway Obstructive Diseases

Therapies that target NF-κB activation or function are potential treatments for GC-refractory airway disease as they act upstream to the actions of GR. However, there may be unintended consequences on immune cell populations by targeting the ubiquitously expressed NF-κB. For example, NF-κB signalling is important in neutrophil apoptosis, which is required for inflammation resolution [49]. The involvement of NF-κB in neutrophil survival may be another explanation for the differing effects of NF-κB inhibitors in models of COPD [42,43,44]. Another approach to treat steroid refractory chronic airway disease is to reverse defects in histone and GR acetylation [87].

### 6.1. Inhibitors of IKK and NF-κB

**Figure 1:**
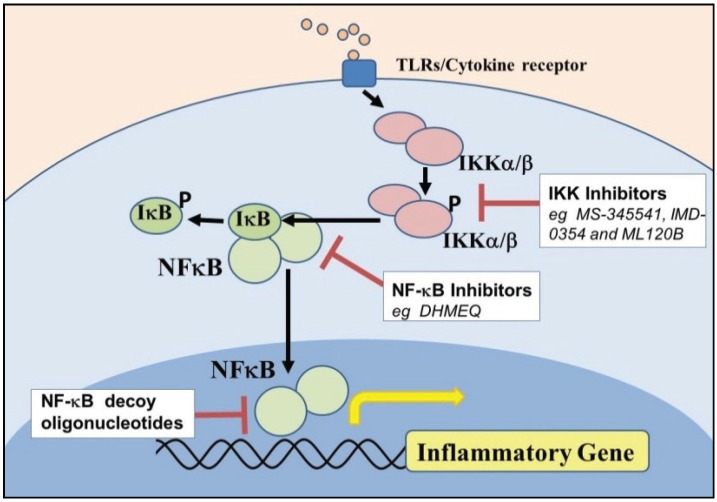
Direct targets of nuclear factor (NF)-kappaB (NF-κB) signaling.

As IκB kinases (IKKs), particularly the β-form (*i.e.*, IKK-β), are essential in NF-κB signaling, their inhibition is therefore a promising mechanism for intervention in asthma and COPD. A number of small molecule inhibitors of IKK-β have been evaluated in both *in vitro* and *in vivo* models of airway disease. The IKK-β inhibitors PS-1145 and ML120B inhibit IL-1β and TNFα-induced expression of cytokines in human ASM cells *in vitro* to the same degree than the maximally effective concentration of the GC, dexamethasone [59]. In human bronchial epithelial cells, the IKK-β inhibitors, S-1145 and ML120B reduced NF-κB-dependent ICAM-1 transcription and expression in response to IL-1β and TNFα, whereas dexamethasone had little effect on expression [53]. The IKK inhibitor MS-345541 and the NF-κB inhibitor pyrrolidinedithiocarbamate reverse the anti-apoptotic effect of TNF-α on human eosinophils [71]. The NF-κB inhibitor dehydroxymethylepoxyquinomicin (DHMEQ) was recently shown to inhibit eotaxin-1 production by bronchial epithelial cells *in vitro* and eosinophilic airway inflammation including Th2 cytokine production and subsequent AWR *in vivo* [54]. The effects of inhibitors which target NF-κB intermediates (see Figure 1) in models of asthma and COPD *in vivo* are summarized in Table 2.

**Table 2:** A summary of findings from studies that have targeted NF-κB intermediates in rodent experimental models of asthma and chronic obstructive pulmonary disease (COPD).

Inhibitor	Target	Model (Animal and Challenge)	Outcome (as Compared to Vehicle Control Mice Following Challenge)
Compound A	IKKβ	Mouse, allergen (cockroach extract)	Reduced airway inflammation and reactivity [99]
Compound A	IKKβ	Rat, allergen (OVA)	Reduced airway inflammation and reactivity [99]
DHMEQ	NF-κB	Mouse, allergen (OVA)	Reduced Th2 eosinophilic airway inflammation and AWR [54]
GSK 657311A	IKKβ	Mouse, LPS and CS extract	Attenuated LPS-stimulated airway inflammation but had no effect on CS-evoked airway inflammation (despite decreasing NF-κB:DNA binding) [44]
IMD-0354 [N-(3,5-bis-trifluoromethyl-phenyl)-5-chloro-2-hydroxy-benzamide]	IKKβ	Mouse, allergen (OVA)	Reduced Th2 eosinophilic airway inflammation [100]
IMD-0354	IKKβ	Mouse, allergen (HDM)	Reduced airway IL-13 production and AWR [101]
NF-κB decoy oligonucleotides	NF-κB	Mouse, CS extract	Decreased lung macrophage number but had no effect on other parameters examined (e.g., TNF-α levels) [42]
PHA-408 [8-(5-chloro-2-(4-methylpiperazin-1-yl)isonicotinamido)-1-(4-fluorophenyl)-4,5-dihydro-1H-benzo[gamma]indazole-3-carboxamide]	IKKβ	Rat, CS extract and LPS	Reduced airway levels of IL-6, TNF-α, IL-1β and GM-CSF [43]
PF-184 [8-(2-(3,4-bis(hydroxymethyl)-3,4-dimethylpyrrolidin-1-yl)-5-chloroisonicotinamido)-1-(4-fluorophenyl)-4,5-dihydro-1H-benzo-[g]indazole-3-carboxamide)]	IKKβ	Rat, LPS	Intratracheal administered PF-184 reduced airway inflammatory cell infiltration and cytokine levels [102]
TPCA-1	IKKβ	Mouse, LPS and CS extract	Attenuated LPS-stimulated airway inflammation but had no effect on CS-evoked airway inflammation (despite decreasing NF-κB:DNA binding) [44]

### 6.2. Indirect Modulators of NF-κB Signaling

A number of approaches reverse the post-translational modifications to histones/GR which negate the transrepression of NF-κB by GCs. Recently, the novel antimicrobial macrolide, solithromycin (CEM-101), was shown to restore GC sensitivity in PBMCs from COPD patients with much greater efficacy than macroglides in current clinical use (e.g., azithromycin) [103]. Solithromycin targets protein phosphatase PP2A, a positive regulator of Akt/PI3K activity, which in turn negatively regulates HDAC2 activity by phosphorylation. Theophylline, formoterol (a long acting β-agonist) and nortriptyline (a tricyclic antidepressant) also inhibit Akt/PI3K and overcome GC-insensitive inflammatory responses in PBMCs from COPD patients and/or CS-extract exposed mice *in vivo* [104,105,106]. All selectively inhibit oxidant-activated PI3K-δ, an isoform of PI3K which is up-regulated in the lungs of COPD patients. Drugs which target HDACs, hence NF-κB signaling, may have beneficial effects in treating airway inflammation independent of GC restoration. Antimicrobial macroglides are used to treat COPD exacerbation and have been shown to inhibit NF-κB signaling in models of COPD independent of GCs [107,108]. Selective inhibitors of PI3K-δ including IC87114 and anthraquinone derivatives also inhibit Th2-driven inflammation in murine models of allergic asthma [109,110,111,112]. Targeting activated PI3K-δ in airway inflammation is a promising treatment for asthma and COPD as this isoform appears to be expressed mainly by leukocytes. PI3K-δ inhibition would be expected to inhibit GC-insensitive responses such as mast cell degranulation and B-cell IgE production, as well as blocking the effects of IL-17, which is implicated in steroid-insensitive severe asthma.

Another approach to target NF-κB signaling is to restore sirtuin activity, which is reduced in chronic respiratory disease. There are synthetic and naturally occurring compounds including polyphenols (*i.e.*, resveratrol, epigallocatechin gallate, quercetin) available which can augment Sirt1 activity. The Sirt1 activator, SRT1720 and resveratrol have been shown in both ovalbumin and HDM models of allergic airway inflammation to have protective effects [113,114,115,116]. Whether the inhibitory effects of SRT1720 and resveratrol on Th2-driven airway inflammation involves increased histone, GR or NF-κB deacetylation requires further investigation. Recently it was shown Sirt1 activation by SRT1720, reduced stress-induced premature cellular senescence and emphysema induced by CS extract and elastase in mice. However, the effects of augmented Sirt1 in these models were attributed to Sirt1-mediated deacetylation of the FOXO3 transcription factor, rather post-translational modifications to NF-κB/IKK [117,118].

## 7. Conclusions

NF-κB has a central role in airway inflammation in asthma and COPD. NF-κB activation is stimulated by cytokines and TLR activation in multiple airway cells types, in turn regulating the expression of immunomodulatory and inflammatory mediators. Many NF-κB-regulated responses are insensitive to the actions of GCs in the disease state. Targeting NF-κB signaling or GC-mediated transrepression of NF-κB are potential interventions for steroid-refractory airway disease.

## References

[1] WHO (2007). Global Surveillance, Prevention and Control of Chronic Respiratory Diseases: A Comprehensive Approach.

[2] Decramer M., Janssens W., Miravitlles M. (2012). Chronic obstructive pulmonary disease. Lancet.

[3] Ambrosino N., Paggiaro P. (2012). The management of asthma and chronic obstructive pulmonary disease: Current status and future perspectives. Expert Rev. Respir. Med..

[4] Tetley T.D. (2005). Inflammatory cells and chronic obstructive pulmonary disease. Curr. Drug Targets Inflamm. Allergy.

[5] Tubby C., Harrison T., Todd I., Fairclough L. (2011). Immunological basis of reversible and fixed airways disease. Clin. Sci..

[6] Papiris S.A., Kollintza A., Karatza M., Manali E.D., Sotiropoulou C., Milic-Emili J., Roussos C., Daniil Z. (2007). CD8^+^ T lymphocytes in bronchoalveolar lavage in idiopathic pulmonary fibrosis. J. Inflamm..

[7] Durham A., Adcock I.M., Tliba O. (2011). Steroid resistance in severe asthma: Current mechanisms and future treatment. Curr. Pharm. Des..

[8] Salter M., Biggadike K., Matthews J.L., West M.R., Haase M.V., Farrow S.N., Uings I.J., Gray D.W. (2007). Pharmacological properties of the enhanced-affinity glucocorticoid fluticasone furoate *in vitro* and in an *in vivo* model of respiratory inflammatory disease. Am. J. Physiol. Lung Cell. Mol. Physiol..

[9] Ghosh S., May M.J., Kopp E.B. (1998). NF-κB and Rel proteins: Evolutionarily conserved mediators of immune responses. Annu. Rev. Immunol..

[10] Hinz M., Scheidereit C. (2014). The IκB kinase complex in NF-κB regulation and beyond. EMBO Rep..

[11] Koziol-White C.J., Panettieri R.A. (2011). Airway smooth muscle and immunomodulation in acute exacerbations of airway disease. Immunol. Rev..

[12] Ward J.E., Harris T., Bamford T., Mast A., Pain M.C., Robertson C., Smallwood D., Tran T., Wilson J., Stewart A.G. (2008). Proliferation is not increased in airway myofibroblasts isolated from asthmatics. Eur. Respir. J..

[13] Flavell S.J., Hou T.Z., Lax S., Filer A.D., Salmon M., Buckley C.D. (2008). Fibroblasts as novel therapeutic targets in chronic inflammation. Br. J. Pharmacol..

[14] Li M., Riddle S.R., Frid M.G., el Kasmi K.C., McKinsey T.A., Sokol R.J., Strassheim D., Meyrick B., Yeager M.E., Flockton A.R. (2011). Emergence of fibroblasts with a proinflammatory epigenetically altered phenotype in severe hypoxic pulmonary hypertension. J. Immunol..

[15] Edwards M.R., Bartlett N.W., Clarke D., Birrell M., Belvisi M., Johnston S.L. (2009). Targeting the NF-κB pathway in asthma and chronic obstructive pulmonary disease. Pharmacol. Ther..

[16] Rajendrasozhan S., Yang S.R., Edirisinghe I., Yao H., Adenuga D., Rahman I. (2008). Deacetylases and NF-κB in redox regulation of cigarette smoke-induced lung inflammation: Epigenetics in pathogenesis of COPD. Antioxid. Redox Signal..

[17] Barnes P.J. (1998). Anti-inflammatory actions of glucocorticoids: Molecular mechanisms. Clin. Sci..

[18] Kagoshima M., Ito K., Cosio B., Adcock I.M. (2003). Glucocorticoid suppression of nuclear factor-κB: A role for histone modifications. Biochem. Soc. Trans..

[19] Adcock I.M., Ito K., Barnes P.J. (2005). Histone deacetylation: An important mechanism in inflammatory lung diseases. COPD.

[20] To T., Stanojevic S., Moores G., Gershon A.S., Bateman E.D., Cruz A.A., Boulet L.P. (2012). Global asthma prevalence in adults: Findings from the cross-sectional world health survey. BMC Public Health.

[21] Buist A.S., McBurnie M.A., Vollmer W.M., Gillespie S., Burney P., Mannino D.M., Menezes A.M., Sullivan S.D., Lee T.A., Weiss K.B. (2007). International variation in the prevalence of COPD (the BOLD Study): A population-based prevalence study. Lancet.

[22] Brusselle G., Bracke K. (2014). Targeting immune pathways for therapy in asthma and chronic obstructive pulmonary disease. Ann. Am. Thorac. Soc..

[23] Hart L.A., Krishnan V.L., Adcock I.M., Barnes P.J., Chung K.F. (1998). Activation and localization of transcription factor, nuclear factor-κB, in asthma. Am. J. Respir. Crit. Care Med..

[24] Gagliardo R., Chanez P., Mathieu M., Bruno A., Costanzo G., Gougat C., Vachier I., Bousquet J., Bonsignore G., Vignola A.M. (2003). Persistent activation of nuclear factor-κB signaling pathway in severe uncontrolled asthma. Am. J. Respir. Crit. Care Med..

[25] Tully J.E., Hoffman S.M., Lahue K.G., Nolin J.D., Anathy V., Lundblad L.K., Daphtary N., Aliyeva M., Black K.E., Dixon A.E. (2013). Epithelial NF-κB orchestrates house dust mite-induced airway inflammation, hyperresponsiveness, and fibrotic remodeling. J. Immunol..

[26] Poynter M.E., Cloots R., van Woerkom T., Butnor K.J., Vacek P., Taatjes D.J., Irvin C.G., Janssen-Heininger Y.M. (2004). NF-κB activation in airways modulates allergic inflammation but not hyperresponsiveness. J. Immunol..

[27] Poynter M.E., Irvin C.G., Janssen-Heininger Y.M. (2002). Rapid activation of nuclear factor-κB in airway epithelium in a murine model of allergic airway inflammation. Am. J. Pathol..

[28] Poynter M.E., Irvin C.G., Janssen-Heininger Y.M. (2003). A prominent role for airway epithelial NF-κB activation in lipopolysaccharide-induced airway inflammation. J. Immunol..

[29] Quinton L.J., Jones M.R., Simms B.T., Kogan M.S., Robson B.E., Skerrett S.J., Mizgerd J.P. (2007). Functions and regulation of NF-κB RelA during pneumococcal pneumonia. J. Immunol..

[30] Donovan C.E., Mark D.A., He H.Z., Liou H.C., Kobzik L., Wang Y., de Sanctis G.T., Perkins D.L., Finn P.W. (1999). NF-κB/Rel transcription factors: c-Rel Promotes airway hyperresponsiveness and allergic pulmonary inflammation. J. Immunol..

[31] Li X., Chen Q., Chu C., You H., Jin M., Zhao X., Zhu X., Zhou W., Ji W. (2014). Ovalbumin-induced experimental allergic asthma is Toll-like receptor 2 dependent. Allergy Asthma Proc..

[32] Lam D., Ng N., Lee S., Batzer G., Horner A.A. (2008). Airway house dust extract exposures modify allergen-induced airway hypersensitivity responses by TLR4-dependent and independent pathways. J. Immunol..

[33] Wilson S.J., Wallin A., Della-Cioppa G., Sandstrom T., Holgate S.T. (2001). Effects of budesonide and formoterol on NF-κB, adhesion molecules, and cytokines in asthma. Am. J. Respir. Crit. Care Med..

[34] Hancox R.J., Stevens D.A., Adcock I.M., Barnes P.J., Taylor D.R. (1999). Effects of inhaled beta agonist and corticosteroid treatment on nuclear transcription factors in bronchial mucosa in asthma. Thorax.

[35] Hart L., Lim S., Adcock I., Barnes P.J., Chung K.F. (2000). Effects of inhaled corticosteroid therapy on expression and DNA-binding activity of nuclear factor kappaB in asthma. Am. J. Respir. Crit. Care Med..

[36] Di Stefano A., Caramori G., Oates T., Capelli A., Lusuardi M., Gnemmi I., Ioli F., Chung K.F., Donner C.F., Barnes P.J. (2002). Increased expression of nuclear factor-κB in bronchial biopsies from smokers and patients with COPD. Eur. Respir. J..

[37] Caramori G., Romagnoli M., Casolari P., Bellettato C., Casoni G., Boschetto P., Chung K.F., Barnes P.J., Adcock I.M., Ciaccia A. (2003). Nuclear localisation of p65 in sputum macrophages but not in sputum neutrophils during COPD exacerbations. Thorax.

[38] Szulakowski P., Crowther A.J., Jimenez L.A., Donaldson K., Mayer R., Leonard T.B., MacNee W., Drost E.M. (2006). The effect of smoking on the transcriptional regulation of lung inflammation in patients with chronic obstructive pulmonary disease. Am. J. Respir. Crit. Care Med..

[39] Gagliardo R., Chanez P., Profita M., Bonanno A., Albano G.D., Montalbano A.M., Pompeo F., Gagliardo C., Merendino A.M., Gjomarkaj M. (2011). IκB kinase-driven nuclear factor-κB activation in patients with asthma and chronic obstructive pulmonary disease. J. Allergy Clin. Immunol..

[40] Brown V., Elborn J.S., Bradley J., Ennis M. (2009). Dysregulated apoptosis and NFκB expression in COPD subjects. Respir. Res..

[41] Yang S.R., Yao H., Rajendrasozhan S., Chung S., Edirisinghe I., Valvo S., Fromm G., McCabe M.J., Sime P.J., Phipps R.P. (2009). RelB is differentially regulated by IκB Kinase-α in B cells and mouse lung by cigarette smoke. Am. J. Respir. Cell Mol. Biol..

[42] Li Y.T., He B., Wang Y.Z., Wang J. (2009). Effects of intratracheal administration of nuclear factor-κB decoy oligodeoxynucleotides on long-term cigarette smoke-induced lung inflammation and pathology in mice. Respir. Res..

[43] Rajendrasozhan S., Hwang J.W., Yao H., Kishore N., Rahman I. (2010). Anti-inflammatory effect of a selective IκB kinase-beta inhibitor in rat lung in response to LPS and cigarette smoke. Pulm. Pharmacol. Ther..

[44] Rastrick J.M., Stevenson C.S., Eltom S., Grace M., Davies M., Kilty I., Evans S.M., Pasparakis M., Catley M.C., Lawrence T. (2013). Cigarette smoke induced airway inflammation is independent of NF-κB signalling. PLoS ONE.

[45] Yao H., Edirisinghe I., Rajendrasozhan S., Yang S.R., Caito S., Adenuga D., Rahman I. (2008). Cigarette smoke-mediated inflammatory and oxidative responses are strain-dependent in mice. Am. J. Physiol. Lung Cell. Mol. Physiol..

[46] Das J., Chen C.H., Yang L., Cohn L., Ray P., Ray A. (2001). A critical role for NF-κB in GATA3 expression and TH2 differentiation in allergic airway inflammation. Nat. Immunol..

[47] Coward W.R., Sagara H., Wilson S.J., Holgate S.T., Church M.K. (2004). Allergen activates peripheral blood eosinophil nuclear factor-κB to generate granulocyte macrophage-colony stimulating factor, tumour necrosis factor-α and interleukin-8. Clin. Exp. Allergy.

[48] Wong C.K., Wang C.B., Li M.L., Ip W.K., Tian Y.P., Lam C.W. (2006). Induction of adhesion molecules upon the interaction between eosinophils and bronchial epithelial cells: Involvement of p38 MAPK and NF-κB. Int. Immunopharmacol..

[49] Langereis J.D., Raaijmakers H.A., Ulfman L.H., Koenderman L. (2010). Abrogation of NF-κB signaling in human neutrophils induces neutrophil survival through sustained p38-MAPK activation. J. Leukoc. Biol..

[50] Murugan V., Peck M.J. (2009). Signal transduction pathways linking the activation of alveolar macrophages with the recruitment of neutrophils to lungs in chronic obstructive pulmonary disease. Exp. Lung Res..

[51] Yang S.R., Chida A.S., Bauter M.R., Shafiq N., Seweryniak K., Maggirwar S.B., Kilty I., Rahman I. (2006). Cigarette smoke induces proinflammatory cytokine release by activation of NF-κB and posttranslational modifications of histone deacetylase in macrophages. Am. J. Physiol. Lung Cell. Mol. Physiol..

[52] Lee H.C., Ziegler S.F. (2007). Inducible expression of the proallergic cytokine thymic stromal lymphopoietin in airway epithelial cells is controlled by NFκB. Proc. Natl. Acad. Sci. USA.

[53] Newton R., Holden N.S., Catley M.C., Oyelusi W., Leigh R., Proud D., Barnes P.J. (2007). Repression of inflammatory gene expression in human pulmonary epithelial cells by small-molecule IκB kinase inhibitors. J. Pharmacol. Exp. Ther..

[54] Shimizu K., Konno S., Ozaki M., Umezawa K., Yamashita K., Todo S., Nishimura M. (2012). Dehydroxymethylepoxyquinomicin (DHMEQ), a novel NF-κB inhibitor, inhibits allergic inflammation and airway remodelling in murine models of asthma. Clin. Exp. Allergy.

[55] Cao J., Ren G., Gong Y., Dong S., Yin Y., Zhang L. (2011). Bronchial epithelial cells release IL-6, CXCL1 and CXCL8 upon mast cell interaction. Cytokine.

[56] Redhu N.S., Saleh A., Halayko A.J., Ali A.S., Gounni A.S. (2011). Essential role of NF-κB and AP-1 transcription factors in TNF-α-induced TSLP expression in human airway smooth muscle cells. Am. J. Physiol. Lung Cell. Mol. Physiol..

[57] Tirumurugaan K.G., Kang B.N., Panettieri R.A., Foster D.N., Walseth T.F., Kannan M.S. (2008). Regulation of the CD38 promoter in human airway smooth muscle cells by TNF-α and dexamethasone. Respir. Res..

[58] Lee C.W., Lin W.N., Lin C.C., Luo S.F., Wang J.S., Pouyssegur J., Yang C.M. (2006). Transcriptional regulation of VCAM-1 expression by tumor necrosis factor-alpha in human tracheal smooth muscle cells: Involvement of MAPKs, NF-κB, p300, and histone acetylation. J. Cell. Physiol..

[59] Catley M.C., Sukkar M.B., Chung K.F., Jaffee B., Liao S.M., Coyle A.J., Haddad El-B., Barnes P.J., Newton R. (2006). Validation of the anti-inflammatory properties of small-molecule IκB Kinase (IKK)-2 inhibitors by comparison with adenoviral-mediated delivery of dominant-negative IKK1 and IKK2 in human airways smooth muscle. Mol. Pharmacol..

[60] John A.E., Zhu Y.M., Brightling C.E., Pang L., Knox A.J. (2009). Human airway smooth muscle cells from asthmatic individuals have CXCL8 hypersecretion due to increased NF-κB p65, C/EBP β, and RNA polymerase II binding to the CXCL8 promoter. J. Immunol..

[61] Alrashdan Y.A., Alkhouri H., Chen E., Lalor D.J., Poniris M., Henness S., Brightling C.E., Burgess J.K., Armour C.L., Ammit A.J. (2012). Asthmatic airway smooth muscle CXCL10 production: Mitogen-activated protein kinase JNK involvement. Am. J. Physiol. Lung Cell. Mol. Physiol..

[62] Gras D., Chanez P., Vachier I., Petit A., Bourdin A. (2013). Bronchial epithelium as a target for innovative treatments in asthma. Pharmacol. Ther..

[63] Clarke D.L., Clifford R.L., Jindarat S., Proud D., Pang L., Belvisi M., Knox A.J. (2010). TNFα and IFNγ synergistically enhance transcriptional activation of CXCL10 in human airway smooth muscle cells via STAT-1, NF-κB, and the transcriptional coactivator CREB-binding protein. J. Biol. Chem..

[64] Pelaia G., Vatrella A., Busceti M.T., Gallelli L., Calabrese C., Terracciano R., Maselli R. (2015). Cellular mechanisms underlying eosinophilic and neutrophilic airway inflammation in asthma. Mediat. Inflamm..

[65] Brusselle G.G., Maes T., Bracke K.R. (2013). Eosinophils in the spotlight: Eosinophilic airway inflammation in nonallergic asthma. Nat. Med..

[66] Chien J.W., Lin C.Y., Yang K.D., Lin C.H., Kao J.K., Tsai Y.G. (2013). Increased IL-17A secreting CD4^+^ T cells, serum IL-17 levels and exhaled nitric oxide are correlated with childhood asthma severity. Clin. Exp. Allergy.

[67] Chesne J., Braza F., Mahay G., Brouard S., Aronica M., Magnan A. (2014). IL-17 in severe asthma. Where do we stand?. Am. J. Respir. Crit. Care Med..

[68] Fujisawa T., Chang M.M., Velichko S., Thai P., Hung L.Y., Huang F., Phuong N., Chen Y., Wu R. (2011). NF-κB mediates IL-1β- and IL-17A-induced MUC5B expression in airway epithelial cells. Am. J. Respir. Cell Mol. Biol..

[69] Dragon S., Hirst S.J., Lee T.H., Gounni A.S. (2014). IL-17A mediates a selective gene expression profile in asthmatic human airway smooth muscle cells. Am. J. Respir. Cell Mol. Biol..

[70] Chang Y., Al-Alwan L., Risse P.A., Halayko A.J., Martin J.G., Baglole C.J., Eidelman D.H., Hamid Q. (2012). Th17-associated cytokines promote human airway smooth muscle cell proliferation. FASEB J..

[71] Kankaanranta H., Ilmarinen P., Zhang X., Adcock I.M., Lahti A., Barnes P.J., Giembycz M.A., Lindsay M.A., Moilanen E. (2014). Tumour necrosis factor-α regulates human eosinophil apoptosis via ligation of TNF-receptor 1 and balance between NF-κB and AP-1. PLoS ONE.

[72] Ordonez C.L., Shaughnessy T.E., Matthay M.A., Fahy J.V. (2000). Increased neutrophil numbers and IL-8 levels in airway secretions in acute severe asthma: Clinical and biologic significance. Am. J. Respir. Crit. Care Med..

[73] Nakagome K., Matsushita S., Nagata M. (2012). Neutrophilic inflammation in severe asthma. Int. Arch. Allergy Immunol..

[74] Pappas K., Papaioannou A.I., Kostikas K., Tzanakis N. (2013). The role of macrophages in obstructive airways disease: Chronic obstructive pulmonary disease and asthma. Cytokine.

[75] Cundall M., Sun Y., Miranda C., Trudeau J.B., Barnes S., Wenzel S.E. (2003). Neutrophil-derived matrix metalloproteinase-9 is increased in severe asthma and poorly inhibited by glucocorticoids. J. Allergy Clin. Immunol..

[76] Wang C.B., Wong C.K., Ip W.K., Li M.L., Tian Y.P., Lam C.W. (2005). Induction of IL-6 in co-culture of bronchial epithelial cells and eosinophils is regulated by p38 MAPK and NF-κB. Allergy.

[77] Kang J.H., Hwang S.M., Chung I.Y. (2015). S100A8, S100A9 and S100A12 activate airway epithelial cells to produce MUC5AC via extracellular signal-regulated kinase and nuclear factor-κB pathways. Immunology.

[78] James A.L., Wenzel S. (2007). Clinical relevance of airway remodelling in airway diseases. Eur. Respir. J..

[79] Amin K., Ludviksdottir D., Janson C., Nettelbladt O., Bjornsson E., Roomans G.M., Boman G., Seveus L., Venge P. (2000). Inflammation and structural changes in the airways of patients with atopic and nonatopic asthma. BHR Group. Am. J. Respir. Crit. Care Med..

[80] Gosselink J.V., Hayashi S., Elliott W.M., Xing L., Chan B., Yang L., Wright C., Sin D., Pare P.D., Pierce J.A. (2010). Differential expression of tissue repair genes in the pathogenesis of chronic obstructive pulmonary disease. Am. J. Respir. Crit. Care Med..

[81] Xia Y.C., Redhu N.S., Moir L.M., Koziol-White C., Ammit A.J., Al-Alwan L., Camoretti-Mercado B., Clifford R.L. (2013). Pro-inflammatory and immunomodulatory functions of airway smooth muscle: Emerging concepts. Pulm. Pharmacol. Ther..

[82] Tran T., Fernandes D.J., Schuliga M., Harris T., Landells L., Stewart A.G. (2005). Stimulus-dependent glucocorticoid-resistance of GM-CSF production in human cultured airway smooth muscle. Br. J. Pharmacol..

[83] Keenan C.R., Salem S., Fietz E.R., Gualano R.C., Stewart A.G. (2012). Glucocorticoid-resistant asthma and novel anti-inflammatory drugs. Drug Discov. Today.

[84] Salem S., Harris T., Mok J.S., Li M.Y., Keenan C.R., Schuliga M.J., Stewart A.G. (2012). Transforming growth factor-β impairs glucocorticoid activity in the A549 lung adenocarcinoma cell line. Br. J. Pharmacol..

[85] Langenbach S.Y., Wheaton B.J., Fernandes D.J., Jones C., Sutherland T.E., Wraith B.C., Harris T., Schuliga M.J., McLean C., Stewart A.G. (2007). Resistance of fibrogenic responses to glucocorticoid and 2-methoxyestradiol in bleomycin-induced lung fibrosis in mice. Can. J. Physiol. Pharmacol..

[86] Bonacci J.V., Schuliga M., Harris T., Stewart A.G. (2006). Collagen impairs glucocorticoid actions in airway smooth muscle through integrin signalling. Br. J. Pharmacol..

[87] Barnes P.J. (2013). Corticosteroid resistance in patients with asthma and chronic obstructive pulmonary disease. J. Allergy Clin. Immunol..

[88] Adcock I.M., Cosio B., Tsaprouni L., Barnes P.J., Ito K. (2005). Redox regulation of histone deacetylases and glucocorticoid-mediated inhibition of the inflammatory response. Antioxid. Redox Signal..

[89] Kagoshima M., Wilcke T., Ito K., Tsaprouni L., Barnes P.J., Punchard N., Adcock I.M. (2001). Glucocorticoid-mediated transrepression is regulated by histone acetylation and DNA methylation. Eur. J. Pharmacol..

[90] Ito K., Yamamura S., Essilfie-Quaye S., Cosio B., Ito M., Barnes P.J., Adcock I.M. (2006). Histone deacetylase 2-mediated deacetylation of the glucocorticoid receptor enables NF-κB suppression. J. Exp. Med..

[91] Rajendrasozhan S., Yang S.R., Kinnula V.L., Rahman I. (2008). SIRT1, an antiinflammatory and antiaging protein, is decreased in lungs of patients with chronic obstructive pulmonary disease. Am. J. Respir. Crit. Care Med..

[92] Chen Y., Wang H., Luo G., Dai X. (2014). SIRT4 inhibits cigarette smoke extracts-induced mononuclear cell adhesion to human pulmonary microvascular endothelial cells via regulating NF-κB activity. Toxicol. Lett..

[93] Keenan C.R., Schuliga M.J., Stewart A.G. (2015). Pro-inflammatory mediators increase levels of the noncoding RNA GAS5 in airway smooth muscle and epithelial cells. Can. J. Physiol. Pharmacol..

[94] Eddleston J., Herschbach J., Wagelie-Steffen A.L., Christiansen S.C., Zuraw B.L. (2007). The anti-inflammatory effect of glucocorticoids is mediated by glucocorticoid-induced leucine zipper in epithelial cells. J. Allergy Clin. Immunol..

[95] Ayroldi E., Migliorati G., Bruscoli S., Marchetti C., Zollo O., Cannarile L., D’Adamio F., Riccardi C. (2001). Modulation of T-cell activation by the glucocorticoid-induced leucine zipper factor via inhibition of nuclear factor κB. Blood.

[96] Che W., Manetsch M., Quante T., Rahman M.M., Patel B.S., Ge Q., Ammit A.J. (2012). Sphingosine 1-phosphate induces MKP-1 expression via p38 MAPK- and CREB-mediated pathways in airway smooth muscle cells. Biochim. Biophys. Acta.

[97] Manetsch M., Che W., Seidel P., Chen Y., Ammit A.J. (2012). MKP-1: A negative feedback effector that represses MAPK-mediated pro-inflammatory signaling pathways and cytokine secretion in human airway smooth muscle cells. Cell Signal..

[98] King E.M., Holden N.S., Gong W., Rider C.F., Newton R. (2009). Inhibition of NF-κB-dependent transcription by MKP-1: Transcriptional repression by glucocorticoids occurring via p38 MAPK. J. Biol. Chem..

[99] Ziegelbauer K., Gantner F., Lukacs N.W., Berlin A., Fuchikami K., Niki T., Sakai K., Inbe H., Takeshita K., Ishimori M. (2005). A selective novel low-molecular-weight inhibitor of IκB kinase-β (IKK-β) prevents pulmonary inflammation and shows broad anti-inflammatory activity. Br. J. Pharmacol..

[100] Sugita A., Ogawa H., Azuma M., Muto S., Honjo A., Yanagawa H., Nishioka Y., Tani K., Itai A., Sone S. (2009). Antiallergic and anti-inflammatory effects of a novel IκB kinase beta inhibitor, IMD-0354, in a mouse model of allergic inflammation. Int. Arch. Allergy Immunol..

[101] Ogawa H., Azuma M., Muto S., Nishioka Y., Honjo A., Tezuka T., Uehara H., Izumi K., Itai A., Sone S. (2011). IκB kinase beta inhibitor IMD-0354 suppresses airway remodelling in a *Dermatophagoides pteronyssinus*-sensitized mouse model of chronic asthma. Clin. Exp. Allergy.

[102] Sommers C.D., Thompson J.M., Guzova J.A., Bonar S.L., Rader R.K., Mathialagan S., Venkatraman N., Holway V.W., Kahn L.E., Hu G. (2009). Novel tight-binding inhibitory factor-κB kinase (IKK-2) inhibitors demonstrate target-specific anti-inflammatory activities in cellular assays and following oral and local delivery in an *in vivo* model of airway inflammation. J. Pharmacol. Exp. Ther..

[103] Kobayashi Y., Wada H., Rossios C., Takagi D., Charron C., Barnes P.J., Ito K. (2013). A novel macrolide/fluoroketolide, solithromycin (CEM-101), reverses corticosteroid insensitivity via phosphoinositide 3-kinase pathway inhibition. Br. J. Pharmacol..

[104] To Y., Ito K., Kizawa Y., Failla M., Ito M., Kusama T., Elliott W.M., Hogg J.C., Adcock I.M., Barnes P.J. (2010). Targeting phosphoinositide-3-kinase-delta with theophylline reverses corticosteroid insensitivity in chronic obstructive pulmonary disease. Am. J. Respir. Crit. Care Med..

[105] Mercado N., To Y., Ito K., Barnes P.J. (2011). Nortriptyline reverses corticosteroid insensitivity by inhibition of phosphoinositide-3-kinase-delta. J. Pharmacol. Exp. Ther..

[106] Rossios C., To Y., Osoata G., Ito M., Barnes P.J., Ito K. (2012). Corticosteroid insensitivity is reversed by formoterol via phosphoinositide-3-kinase inhibition. Br. J. Pharmacol..

[107] Kobayashi Y., Wada H., Rossios C., Takagi D., Higaki M., Mikura S., Goto H., Barnes P.J., Ito K. (2013). A novel macrolide solithromycin exerts superior anti-inflammatory effect via NF-κB inhibition. J. Pharmacol. Exp. Ther..

[108] Li M., Zhong X., He Z., Wen M., Li J., Peng X., Liu G., Deng J., Zhang J., Bai J. (2012). Effect of erythromycin on cigarette-induced histone deacetylase protein expression and nuclear factor-κB activity in human macrophages *in vitro*. Int. Immunopharmacol..

[109] Lee K.S., Park S.J., Kim S.R., Min K.H., Jin S.M., Puri K.D., Lee Y.C. (2006). Phosphoinositide 3-kinase-delta inhibitor reduces vascular permeability in a murine model of asthma. J. Allergy Clin. Immunol..

[110] De Souza Alves C.C., Collison A., Hatchwell L., Plank M., Morten M., Foster P.S., Johnston S.L., da Costa C.F., de Almeida M.V., Couto Teixeira H. (2013). Inhibiting AKT phosphorylation employing non-cytotoxic anthraquinones ameliorates TH2 mediated allergic airways disease and rhinovirus exacerbation. PLoS ONE.

[111] Choi Y.H., Jin G.Y., Li L.C., Yan G.H. (2013). Inhibition of protein kinase C delta attenuates allergic airway inflammation through suppression of PI3K/Akt/mTOR/HIF-1 alpha/VEGF pathway. PLoS ONE.

[112] Kim S.R., Lee K.S., Park S.J., Min K.H., Choe Y.H., Moon H., Yoo W.H., Chae H.J., Han M.K., Lee Y.C. (2010). Involvement of sirtuin 1 in airway inflammation and hyperresponsiveness of allergic airway disease. J. Allergy Clin. Immunol..

[113] Chen J., Zhou H., Wang J., Zhang B., Liu F., Huang J., Li J., Lin J., Bai J., Liu R. (2015). Therapeutic effects of resveratrol in a mouse model of HDM-induced allergic asthma. Int. Immunopharmacol..

[114] Ichikawa T., Hayashi R., Suzuki K., Imanishi S., Kambara K., Okazawa S., Inomata M., Yamada T., Yamazaki Y., Koshimizu Y. (2013). Sirtuin 1 activator SRT1720 suppresses inflammation in an ovalbumin-induced mouse model of asthma. Respirology.

[115] Royce S.G., Dang W., Yuan G., Tran J., el Osta A., Karagiannis T.C., Tang M.L. (2011). Resveratrol has protective effects against airway remodeling and airway hyperreactivity in a murine model of allergic airways disease. Pathobiol. Aging Age Relat. Dis..

[116] Lee M., Kim S., Kwon O.K., Oh S.R., Lee H.K., Ahn K. (2009). Anti-inflammatory and anti-asthmatic effects of resveratrol, a polyphenolic stilbene, in a mouse model of allergic asthma. Int. Immunopharmacol..

[117] Yao H., Chung S., Hwang J.W., Rajendrasozhan S., Sundar I.K., Dean D.A., McBurney M.W., Guarente L., Gu W., Ronty M. (2012). SIRT1 protects against emphysema via FOXO3-mediated reduction of premature senescence in mice. J. Clin. Invest..

[118] Yao H., Sundar I.K., Ahmad T., Lerner C., Gerloff J., Friedman A.E., Phipps R.P., Sime P.J., McBurney M.W., Guarente L. (2014). SIRT1 protects against cigarette smoke-induced lung oxidative stress via a FOXO3-dependent mechanism. Am. J. Physiol. Lung Cell. Mol. Physiol..

